# Schizophrenia hospitalizations - a big data approach

**DOI:** 10.1192/j.eurpsy.2021.425

**Published:** 2021-08-13

**Authors:** M. Gonçalves-Pinho, J.P. Ribeiro, A. Freitas

**Affiliations:** 1 Department Of Psychiatry And Mental Health, Centro Hospitalar do Tâmega e Sousa, Penafiel, Portugal, Penafiel, Portugal; 2 2d4h, Center for Health Technology and Services Research (CINTESIS), Porto, Portugal

**Keywords:** schizophrénia, Big Data, Administrative Database

## Abstract

**Introduction:**

Schizophrenia is characterized by long hospitalizations and a recurrent use of chronic and acute psychiatric care.

**Objectives:**

The aim of this study was to analyze schizophrenia related hospitalizations in Portugal.

**Methods:**

A retrospective observational study was conducted using a nationwide hospitalization database containing all hospitalizations registered in Portuguese public hospitals from 2008 to 2015.Hospitalizations with a primary diagnosis of schizophrenia were selected and schizophrenia subtypes were grouped using the International Classification of Diseases version 9, Clinical Modification(ICD-9-CM) codes of diagnosis 295.xx.

**Results:**

There was a total of 25,385 hospitalizations in public hospitals of Portugal between 2008 and 2015 with a primary diagnosis of Schizophrenia or other psychotic disorders. A total of 14,279 patients were hospitalized during the study period with an average of 1,78 hospitalizations episodes per patient in the 8-year interval(0.22 hospitalizations/patient/year). 68.0% of the hospitalizations occurred in male patients and the median length of stay was 18.0 days. Mean hospitalization charges were 3,509.7€ per hospitalization, summed to a total charge of 89.1M€. Throughout the study period there was a significant linear decrease in the number of hospitalizations (r = 0.940; B= -47.488; p = 0.001). The last year of the study(2015) had the lowest number of hospitalizations with a total of 2,958 (vs. 3,314 in 2008). When adjusted for the yearly population, there was also a decrease of the number of hospitalizations per 100,000 inhabitants from 31.39 to 28.56 hospitalizations per 100,000 inhabitants between 2008 and 2015, respectively.

**Conclusions:**

We found differences in hospitalization characteristics by gender, age and primary diagnosis.

**Disclosure:**

No significant relationships.

